# Role of Pyroptosis in Inflammatory Bowel Disease (IBD): From Gasdermins to DAMPs

**DOI:** 10.3389/fphar.2022.833588

**Published:** 2022-05-23

**Authors:** Shuxia Zhang, Yujie Liang, Jun Yao, De-feng Li, Li-sheng Wang

**Affiliations:** ^1^ School of Medicine, Southern University of Science and Technology, Shenzhen People Hospital, Shenzhen, China; ^2^ Shenzhen Kangning Hospital, Shenzhen, China; ^3^ Department of Gastroenterology, The Second Clinical Medicine College (Shenzhen People’s Hospital), Jinan University, Shenzhen, China

**Keywords:** IBD, pyroptosis, gasdermin, intestinal pathogens, HGMB1

## Abstract

Pyroptosis is a pro-inflammatory cell death executed by gasdermin family proteins that involve the formation of pores on cells, recognition of danger signals, and release of pro-inflammatory cytokines IL-1*β* and IL-18. Pyroptosis modulates mucosal innate immunity and enteropathogenic bacterial infection. Similarly, the gasdermin family has been reported to be involved in the defense of the intestinal epithelium against bacterial infection and in the regulation of intestinal inflammation. Pyroptosis initiates damage signals that activate multiple pathways to cause inflammation, which may be a potential cause of chronic intestinal inflammation. In this review, we discuss the impact of pyroptosis on inflammatory bowel disease (IBD), with a focus on the executive proteins of pyroptosis (GSDMB, GADMD, and GSDME) and IBD-related endogenous damage-associated molecular patterns (DAMPs) produced by pyroptosis.

## Introduction

Intestinal inflammatory disease (IBD) is a chronic bowel disease that has become a global health problem (Kaplan, 2015). IBD primarily consists of Crohn’s disease (CD) and ulcerative colitis (UC), which are chronic, relapsing gastrointestinal conditions characterized by intestinal inflammation and epithelial injury (Neurath, 2014). CD is a chronic systemic inflammatory disease that affects primarily the gastrointestinal tract, as well as displaying extraintestinal manifestations and immune-related disorders (Baumgart and Sandborn, 2012). UC, the most common form of inflammatory bowel disease worldwide, is a mucosal disease that is less prone to complications (Danese and Fiocchi, 2011). IBD is a chronic inflammatory disease with no cure and significant morbidity (Abraham and Cho, 2009). Current treatment methods are typically targeted biological therapies and non-targeted therapies. Biological therapies are effective for many patients; however, up to 30% of patients fail to respond after initial treatment (Chang, 2020). Patients with IBD are at increased risk for gastrointestinal and extra-intestinal malignancies, particularly colorectal cancer (CRC) and lymphoma (Nadeem et al., 2020).

The pathogenesis of IBD is complex, related to epithelial barrier function, innate immunity, and adaptive immunity (Xavier and Podolsky, 2007). In past years, extraordinary progress has been made explicating the pathogenesis of inflammatory bowel disease. Factors responsible for IBD include genetic components, environmental elements, microbial flora, and immune responses (Abraham and Cho, 2009). Population-based studies have found an increased incidence of IBD in newly industrialized countries in Africa, Asia, and South America, while the highest incidence is observed in developed countries in North America, Oceania, and Europe, potentially reflecting the impact of the environment on IBD (Ng et al., 2017). Genome-wide association studies (GWAS) and subsequent meta-analyses have identified at least 163 IBD-associated loci. Reports have also suggested that host-microbe interactions affect IBD (Jostins et al., 2012). Intestinal flora dysfunction is a current hot topic in IBD research. Many studies have found that fecal microbiota in IBD patients is unstable and shows reduced biodiversity compared to healthy controls (Andoh et al., 2011; Joossens et al., 2011).

While significant progress has been made in studying the pathogenesis of IBD, mucosal inflammation remains unaddressed. The intestinal epithelium, an important component of the intestinal barrier, is susceptible to infection by pathogenic bacteria that disrupt intestinal barrier function and cause inflammation. Infected intestinal epithelial cells (IECs) fight pathogenic bacteria through a pro-inflammatory cell death pattern called cell pyroptosis. Pyroptosis is a lytic process characterized by swollen cells, large bubbles blowing from the plasma membrane, and the release of two pro-inflammatory cytokines [interleukin-1*β* (IL-1*β*) and interleukin-18(IL-18)] as a defense against intracellular pathogens (Jorgensen and Miao, 2015). Pyroptosis promotes cell death *via* inflammation, and many studies have identified a role for pyroptosis in IBD (Zhou and Fang, 2019; Patankar and Becker, 2020). However, many claims about the role of pyroptosis in intestinal pathology have been extrapolated from studies of the inflammasome (Rathinam and Chan, 2018; Zheng et al., 2021). Gasdermins (GSDMs) family is abundantly expressed in skin and gastrointestinal epithelium (Shi et al., 2017). The gasdermin family is composed of Gasdermin A (GSDMA), Gasdermin B (GSDMB), Gasdermin C (GSDMC), Gasdermin D (GSDMD), Gasdermin E (GSDME, DFNA5) and Pejvakin (PJVK) (Tamura et al., 2007; Feng et al., 2018), which have N-terminal structural domains capable of inducing cell death in mammalian cells (Shi et al., 2017). For these proteins, particular attention has been paid to mechanisms of pyroptosis promoted by GSDMB, GSDMD, and GSDME. Many studies on the role of pyroptosis in IBD have been focused on the inflammasome and caspase. However, less attention was paid to the role of gasdermin protein as an executive protein of pyroptosis on IBD. Therefore, in this review, we highlight the effects on IBD of the pyroptosis executive gasdermin proteins and associated released substances.

## Mechanisms of Pyroptosis

Pyroptosis is an inflammatory form of cell death (Kayagaki et al., 2015). In the 1980s and 1990s, pyroptosis was thought to be a toxin-stimulated or pathogen-infected cystatin 1-dependent form of macrophage death. Due to the involvement of caspase-1 (involved in apoptosis), pyroptosis was historically referred to as caspase-1-mediated monocyte death (Zychlinsky et al., 1992). However, scientists later discovered that, although both processes involve caspases, cell death *via* apoptosis is not inflammatory, but the newly discovered form of cell death is. This new form of cell death was named ‘pyroptosis’ by Brad Cookson and his coworkers to distinguish proinflammatory apoptosis from the classical process (Cookson and Brennan, 2001). Since then, there were no breakthroughs in pyroptosis research until 2015, when three independent laboratories used different approaches to confirm that GSDMD acts as an effector protein of pyroptosis by caspases-1/4/5/11 (caspase 11 in mice, caspase 4/5 in humans) to trigger cell death (He et al., 2015; Kayagaki et al., 2015; Shi et al., 2015).

Inflammasome response sensors recognize pathogen-associated molecular patterns (PAMPs) and damage-associated molecular patterns (DAMPs), they stimulate the assembly of inflammasome complexes. Inflammasome-activated caspase-1 cleaves GSDMD into two fragments: N-terminal proteolytic fragment of GSDMD (GSDMD^NT^) and C-terminal proteolytic fragment of GSDMD (GSDMD^CT^). GSDMD^NT^ is a key point for pore formation. GSDMD is a key factor in pore formation. The N-terminal and C-terminal of the full-length GSDMD are self-repressed and activated when the GSDMD is cleaved to release GSDMD^NT^ (Shi et al., 2015). GSDMD^NT^ efficiently solubilizes phosphatidylinositol/cardiolipid-containing liposomes and forms pores in membranes made of artificial or natural phospholipid mixtures. The formation of GSDMD membrane pores disrupts the osmotic potential, leading to cell swelling and eventual disintegration (Ding et al., 2016). Besides GSDMD, the n-terminal domains of the gasdermin family (human) members GSDMA, GSDMB, GSDMC, and GSDME all can form pores (Feng et al., 2018).

**FIGURE 1:**
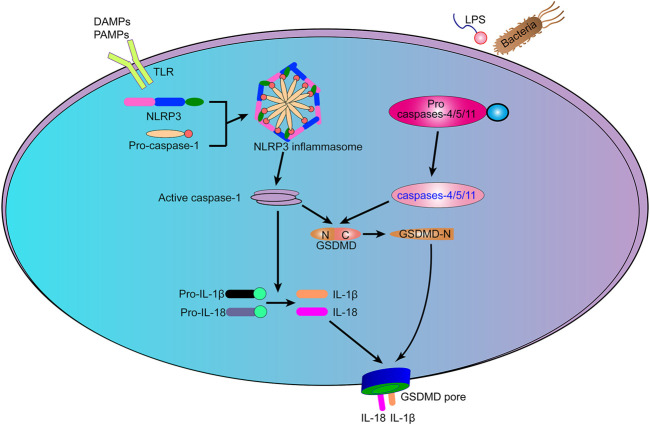
The mechanism of pyroptosis. Typical inflammasomes act as sensors for various types of cell damage signals. The activation of caspases-1/4/5/11 initiates many pathways that promote cell death signaling, causing the pro-inflammatory cytokines IL-1*β* and IL-18 to be cleaved and released through large holes formed by N-terminal proteolytic fragment of GSDMD (GSDMD^NT^) in the plasma membrane and eventually leading to cell death.

Cytokines pro-IL-1*β* and pro-IL-18 are also cleaved to produce mature IL-1*β* and IL-18. GSDMD^NT^ is cytotoxic and plays a role in the formation of pores in the cell membrane (Shi et al., 2017). IL-1*β* and IL-18 are released from the pyroptosis pore (Figure 1). In the absence of GSDMD, the expression of IL-1*β* and IL-18 was not blocked, but the amount secreted extracellularly was reduced, indicating the dependence of IL-1*β* and IL-18 release on focal death (Shi et al., 2015).

As more and more pores are formed, the cell eventually ruptures (Broz et al., 2020). Similar to immune reactions, pyroptosis leads to harmful effects. Pyroptosis plays a role in preventing host infection from pathogenic bacteria and is an important innate immune defense mechanism (Jorgensen and Miao, 2015). However, excessive and uncontrolled pyroptosis may lead to organismal damage, by causing a severe inflammatory response. For example, chimeric antigen receptor (CAR) T cell therapy has become a very effective cancer treatment, but it remains hindered by cytokine release syndrome (CRS). It has been shown that CAR T cells release Granzyme B (GZMB), which triggers GSDME-mediated target cell pyroptosis to cause CRS (Liu et al., 2020).

## Pyroptosis and Intestinal Pathogens

**FIGURE 2:**
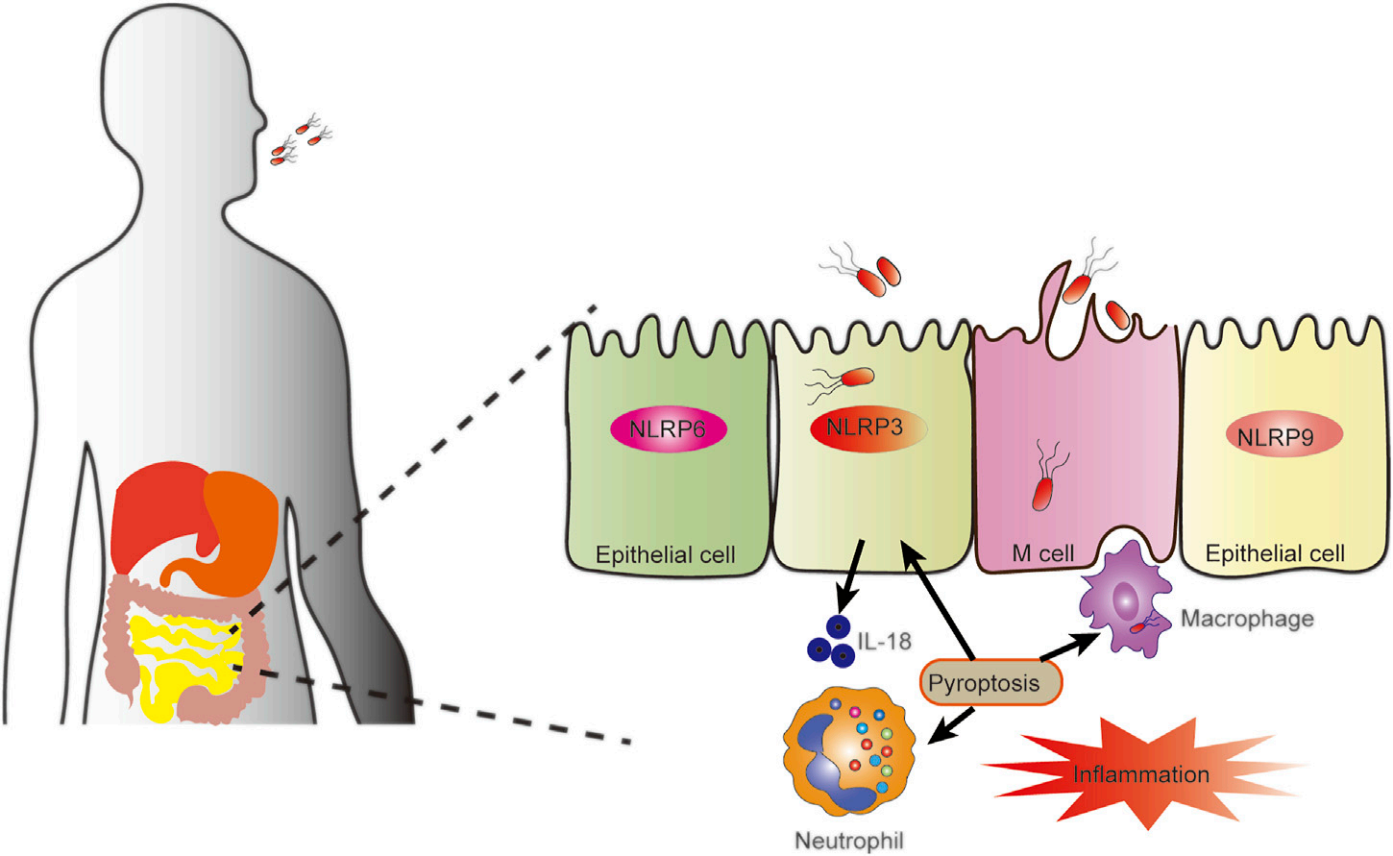
Pyroptosis against intestinal pathogens. Pyroptosis plays a role in interactions between pathogens and intestinal epithelial cells or resident immune cells maintain intestinal homeostasis and inflammation. When pathogens invade epithelial cells, inflammasomes in the epithelial cells, including NLRP3, NLRP6, and NLRP9, are activated to secrete cytokine IL-18. The activation of the inflammasome in epithelial cells can also cause pyroptosis, ultimately leading to the release of bacteria that have invaded epithelial cells, stimulation of the immune response, and enhanced mucosal immune defense.

As a protective host defense, pyroptosis plays a crucial role in controlling several types of viral and bacterial infections (Figure 2) (Jorgensen et al., 2017; Banerjee et al., 2018; Cerqueira et al., 2018; Wang et al., 2019). PAMPs and DAMPs, including lipopolysaccharides (LPS), flagellins, and lipoproteins, are detected by the inflammasome when they infect cells. Different bacterial proteins are recognized by different receptors, which trigger pyroptosis and promote pathogen clearance (Bergsbaken et al., 2009; Cunha and Zamboni, 2013). In different mouse models (DSS, Oxazolone, and TNBS models) NLRP3 has been shown to protect epithelial integrity, regulate intestinal homeostasis, and attenuate experimental colitis (Allen et al., 2010; Zaki et al., 2010; Hirota et al., 2011). Excessive NLRP3 activation, which can be caused by knockdown of V-set and immunoglobulin domain-containing 4 (VSIG4), protects mice from DSS-induced colitis (Huang et al., 2019). However, in other studies, the opposite outcome was reported (Bauer et al., 2010; Bauer et al., 2012). In a DSS-induced colitis model, NLRP3 (−/−) mice were shown to release less IL-1*β*, a central factor in the pathogenesis of IBD, and NLRP3 (−/−) mice were less likely to exhibit colitis than wild-type controls (Bauer et al., 2010). This contradictory result may be explained by differences in intestinal microflora composition (Bauer et al., 2012). In intestinal epithelial cells, NLRP9b is involved in GSDMD-induced pyroptosis to restrict rotavirus infection (Zhu et al., 2017). NLRP6 inhibits enterovirus infection, maintains intestinal flora homeostasis, and regulates antimicrobial immunity (Wang et al., 2015).

Pyroptosis promotes host defense against intracellular bacteria by trapping pathogens in pore-induced intracellular traps (PITs) in pyroptotic macrophages (Jorgensen et al., 2016). Pyroptosis damages the outer membrane of bacteria, but not fatally so. And such living bacteria remain trapped in the cellular debris of the pyroptotic cell. The pyroptotic cell corpse has a plasma membrane that remains largely intact, retaining both organelles and bacteria. This pore-induced cellular debris, or PIT, can capture intracellular bacteria. In addition to capturing bacteria, PIT orchestrates innate immune responses, driving neutrophil recruitment and phagocytosis, with this secondary phagocytosis ultimately killing captured bacteria (Jorgensen et al., 2016).

Pyroptosis plays a role in preventing host infection by pathogenic bacteria (Jorgensen and Miao, 2015). However, invading microorganisms also resist or prohibit the pyroptosis response through complex mechanisms. *Shigella flexneri* (*S. flexneri*) uses multiple strategies to counteract the innate immune system, including escaping immune surveillance by delivering a subset of T3SS effectors during epithelial cell replication (Ashida et al., 2011). Recent studies have identified IpaH7.8, a T3SS effector protein secreted by the intestinal invasive *S. flexneri*, which acts on GSDMB for ubiquitin-mediated proteolysis. Therefore, *S. flexneri* is protected from the bactericidal activity of NK cells by inhibiting GSDMB-mediated pyroptosis induced by Granzyme A (GMZA) (Hansen et al., 2021).

Taken together, these findings define pyroptosis as a pro-inflammatory form of cell death that plays a role in the maintenance of intestinal homeostasis, as well as in intestinal inflammation. The interactions between host and pathogen are complex, with host defense mechanisms preventing pathogens from invading and pathogens enacting corresponding countermeasures to evade the innate immune system.

## Gasdermins in IBD

The gasdermin family is composed of Gasdermin A (GSDMA), Gasdermin B (GSDMB), Gasdermin C (GSDMC), Gasdermin D (GSDMD), Gasdermin E (GSDME, DFNA5), and Pejvakin (PJVK) (Tamura et al., 2007; Feng et al., 2018), Gasdermins (GSDMs) family is abundantly expressed in skin and gastrointestinal epithelium (Shi et al., 2017), which have N-terminal structural domains capable of inducing cell death in mammalian cells (Shi et al., 2017). Of these proteins, particular attention has been paid to mechanisms of pyroptosis promoted by GSDMB, GSDMD, and GSDME. In the mouse gastrointestinal tract, gasdermin genes exhibit tissue-specific linear expression (Tamura et al., 2007). Based on studies of the expression pattern of this family of genes in upper gastrointestinal epithelial cells and cancer, GSDMB demonstrated no inhibition of cell growth in gastric cancer cells and may be an oncogene. GSDMD has been expressed in differentiated cells and may be a potential tumor suppressor (Saeki et al., 2009). Analysis of promoter methylation patterns in colorectal cancer (CRC) cell lines identified GSDME as a tumor suppressor for colon cancer (Yokomizo et al., 2012).

### GSDMB

Katoh discovered GSDMB (GSDML, PP4052, and PRO2521) in 2004 (Katoh and Katoh, 2004). GSDMB, unlike other members of the gasdermin family, is not found in mice, although it is present in humans and other mammals (Tamura et al., 2007). GSDMB is highly expressed in the skin epithelium, gastrointestinal tract, and immune cells (17q12-q21 locus) (Saeki et al., 2009). GSDMB, a member of the gasdermin family of executive pyroptosis proteins, has been found to play a role in pyroptosis. Studies have demonstrated that the Gasdermin-N domain of GSDMB, although not the full-length GSDMB protein, can induce extensive pyroptosis in 293T cells (Ding et al., 2016).

GSDMB is cleaved by caspase-1 at the aspartate 236 position relative to the GSDMB N-terminus and released to induce potent pyroptosis. In contrast, by mutating the aspartate of residue 236 to alanine (when D236A), pyroptosis does not occur (Panganiban et al., 2018). Apoptotic caspase-3/6/7 also cleaves GSDMB, producing p36 and p10 fragments (Chao et al., 2017). Recent studies have found that gasdermin cleavage is not caspase-specific. GSDMB also promotes caspase-4 activity, which is required for the cleavage of GSDMD in non-canonical pyroptosis (Chen et al., 2019a). Shaofeng et al. found that GSDMB expressed in human embryonic kidney (HEK) 239T cells could be specifically cleaved into GSDMB^NT^ and GSDMB^CT^ by GZMA, which is secreted by NK cells and cytotoxic T cells. Similarly, GSDMB^NT^ acts on the cell membrane to promote pore formation, after which some molecules are expelled from the pore, and the cell swells, causing pyroptosis (Zhou et al., 2020).

GWAS has identified several autoimmune diseases associated with GSDMB, including asthma (Wu et al., 2009; Li et al., 2020), type 1 diabetes (Saleh et al., 2011), IBD (Söderman et al., 2015; Chao et al., 2017), and rheumatoid arthritis. GWAS has also shown correlations between IBD susceptibility and single nucleotide polymorphisms (SNPs) in the protein-coding and transcriptional regulatory regions of GSDMB (Das et al., 2017). Based on the study of gene expression levels in samples from IBD patients (inflammatory and non-inflammatory mucosa) and non-IBD patients (non-inflammatory mucosa), GSDMB was suggested to contribute to IBD susceptibility (Söderman et al., 2015). To further support this GWAS data, a structural analysis of GSDMB was performed to explore potential disease mechanisms (Chao et al., 2017). Immunohistochemical analysis of colon tissues showed that GSDMB expression levels were higher in Crohn’s disease samples than in normal colon tissues. It suggests that there may be a relationship between GSDMB and IBD and that this relationship may be that GSDMB regulates intestinal inflammation by promoting GSDMD-mediated pyrosis (Chen et al., 2019a). Many studies have confirmed that the GSDMB gene is associated with IBD susceptibility. However, insufficient research has explored the mechanisms by which GSDMB affects IBD, and it remains uncertain whether GSDMB-induced pyroptosis can contribute to the development of intestinal inflammation.

### GSDMD

GSDMD is predominantly expressed in the small intestine, but weak expression has also been reported in the colon and stomach (Tamura et al., 2007). GSDMD was one of the first members of the gasdermin family to be identified as a pyroptosis-executing protein (Shi et al., 2015), and it remains the most studied pyroptosis execution protein. Molecular structures of mouse and human GSDMD were reported in 2019 (Liu et al., 2019a).

In bone-marrow-derived macrophages (iBMDMs), GSDMD is cleaved by inflammatory caspase-1 after the 272FLTD275 (or 273LLSD276) sequence in response to LPS stimulation and activation of the canonical inflammasome pathway (Shi et al., 2015). Full-GSDMD is cleaved into cytotoxic GSDMD^NT^ and GSDMD^CT^. GSDMD^NT^ binds to phosphatidylinositol phosphates and phosphatidylserine (restricted to the inner leaflet of the cell membrane), as well as to cardiolipin (present in both inner and outer leaflets of bacterial membranes) (Broz et al., 2020). GSDMD^NT^ binds with lipid leading to pyroptosis, but it does not damage neighboring cells (Liu et al., 2016). Noncanonical inflammasome signaling through caspase-11 in mice and caspase-4/5 in humans activates pyroptosis (Hagar et al., 2013; Kayagaki et al., 2013). GSDMD can also be cleaved by the apoptotic caspase (caspase-8) (Orning et al., 2018). In addition to cleavage by caspase (caspase-1/4/5/8/11), GSDMD can also be cleaved by neutrophil elastase and cathepsin G (Sollberger et al., 2018; Burgener et al., 2019; Bulek et al., 2020). Although the cleavage site is different from that utilized by caspases, the GSDMD^NT^ product can still promote pore formation. For these reasons, pyroptosis is considered to be a gasdermin-mediated necrotic form of programmed cell death (Shi et al., 2017; Galluzzi et al., 2018).

Pyroptosis protects the host from pathogenic infection through cell lysis and is critical to innate immunity. For example, GSDMD-deficient mice are more susceptible to rotavirus infection than wild-type mice (Zhu et al., 2017). A similar phenomenon has been found again. Mice with GSDMD-deficient BMDMs are more vulnerable to *Francisella novicida* (*F. novicida*, intracellular bacteria) infection than WT mice (Banerjee et al., 2018). Indeed, GSDMD is required for host defense against *F. novicida* infection (Zhu et al., 2018). In the DSS-induced colitis mouse model, overgrowth of commensal *E. coli*, which mediated the activation of GSDMD in IEC (GSDMD was strongly cleaved to activated GSDMD^NT^) promoted pyroptosis. In contrast, the severity of colitis was attenuated in mice deficient in GSDMD (Gao et al., 2021). In response to challenges by DAMPs and PAMPs, GSDMD-mediated pyroptosis promotes inflammation. However, it has been reported that GSDMD also exerts anti-inflammatory functions by controlling neutrophil death. Elastase-induced neutrophil death negatively regulates neutrophil-mediated innate immunity and exerts anti-inflammatory effects. Following infection by *Escherichia coli* (*E. coli*), GSDMD deficiency enhances antimicrobial activity by increasing neutrophil survival (Kambara et al., 2018). NLRP9b recognizes rotavirus RNA pathogen-associated molecular patterns in intestinal epithelial cells and promotes GSDMD-induced pyroptosis in IECs (Zhu et al., 2017). Thus, the interaction between pyroptosis and inflammation depends on the responding host cell as well as, potentially, on the type of pathogen.

Patients with IBD and experimental colitis exhibit increased expression of epithelial-derived GSDMD, suggesting a potential role for GSDMD in IBD (Bulek et al., 2020). Evidence also suggests that GSDMD plays a protective role in DSS-induced colitis (Gao et al., 2021). GSDMD deficiency-induced inflammation in macrophages that aggravated experimental colitis by boosting cyclic GMP-AMP synthase (cGAS)-dependent inflammation (Ma et al., 2020). GSDMD-induced pyroptosis leads to the formation of pores in the cell membrane, giving rise to ionic fluxes (Rühl and Broz, 2015). Efflux of potassium (K^+^) has been identified as particularly critical, as K^+^ loss from the cell inhibits cytosolic DNA (DAMP)-induced cGAS signaling (Banerjee et al., 2018). In DSS-induced colitis models, DSS disrupts the intestinal epithelial monolayer lining, allowing intestinal pathogens to invade the mucosa and provoking an acute inflammatory response in the lamina propria (Eichele and Kharbanda, 2017). Furthermore, extracellular bacteria and dying cells can both release CDNs (cyclic dinucleotides), which activate cGAS-STING signaling (Liu et al., 2019b). cGAS-STING signaling activates transcription factors IRF3 and nuclear factor κB, thereby inducing the production of type I IFNs and other cytokines capable of triggering inflammatory responses (Cai et al., 2014). Activation of cGAS-STING signaling is responsible for the exacerbation of intestinal inflammation (Banerjee et al., 2018). NIMA-associated kinase 7 (NEK7) is an NLRP3-binding protein that interacts with the leucine-rich repeat domain of NLRP3. When NEK7 is knocked down, the activation of caspase-1 and release of IL-1*β* are restricted (He et al., 2016). The mRNA expression of NEK7, Caspase-1, NLRP3 and GSDMD was significantly higher in UC tissues than in the control group. In the DSS-induced mouse model, knockdown of NEK7 was associated with reduced protein expression levels of Caspase-1, NLRP3, and GSDMD. This suggests that NEK7-NLRP3 interaction regulation GSDMD-mediates pyroptosis may be a novel mechanism for IBD (Chen et al., 2019b). In a DSS-induced mouse colitis model, the use of VX765 (a caspase-1 inhibitor), which inhibits caspase-1/GSDMD pathway-regulated pyroptosis, attenuates colitis in mice (Wang et al., 2022). The 18-kDa translocator protein (TSPO) is one of the outer mitochondrial membrane proteins (Wang et al., 2016). Gly-Pro-Ala (GPA), a GPA peptide isolated from fish skin gelatin hydrolysate (Zheng et al., 2018), ameliorate DSS-induced colitis by inhibiting GSDMD-mediates pyroptosis (Deng et al., 2020). These examples above illustrate that GSDMD mediated pyroptosis and IBD are closely related and can be regulated by modulating GSDMD-mediated pyroptosis in a DSS-induced colitis model.

Although GSDMD is more highly expressed in epithelial cells than in lamina propria hematopoietic cells, GSDMD in IECs did not protect against damage caused by DSS treatment in mice (Maik-Rachline et al., 2020; Gao et al., 2021). The protective effect of GSDMD in macrophages against DSS-induced colitis has been demonstrated (Ma et al., 2020). So, what is the role of high GSDMD expression in IEC? Previous studies have reported that GSDMD is more highly expressed in the IECs of IBD patients compared to healthy individuals. Full-length GSDMD plays a non-pyroptotic role in promoting the release of IL-1*β*-containing small extracellular vesicles (sEVs) from IECs (Bulek et al., 2020). Interestingly, studies have also reported that GSDMD^NT^ released from pyroptotic cells kills pathogens (Liu et al., 2016). Direct killing of intracellular bacteria by GSDMD^NT^ limits the release of viable bacteria from pyroptotic cells and reduces infection. Taken together, the literature suggests that GSDMD in IECs may protect against intestinal inflammation through non-pyroptotic means.

Recently, it has also been shown that Caspase-8-GSDMD triggers pyroptosis-like cell death of IECs in FADD-deficient mice, contributing to the development of intestinal inflammation (Schwarzer et al., 2020). This mechanism may be related to the pathogenesis of intestinal inflammation during intestinal pathogenic bacterial infections. Enteropathogenic bacteria express effectors that target FADD, including arginine glycosyltransferase NleB1 (Scott et al., 2017), and inhibit its function. Caspase-8 promotes intestinal inflammation in FADD-dysfunctional mice by inducing the death of GSDMD-mediated IECs (Schwarzer et al., 2020).

The relationship between GSDMD and intestinal inflammation is complex, dependent on both the type of infecting pathogen and the host cells. Current studies in macrophages found that GSDMD causes cellular K loss by triggering pyroptosis, which in turn inhibits cGAS-STING signaling to protect the host and alleviate inflammation. In IECs, GSDMD guides the extracellular release of IL-1*β* through non-pyroptosis to exacerbate inflammation. It has also been shown that GSDMD induces the release of IL-18, but not IL-1*β*, to stimulate inflammation. In FADD knockout mice, GSDMD caused intestinal inflammation due to lytic death of IECs (Schwarzer et al., 2020), a phenomenon that may explain the inflammation caused by some intestinal pathogenic bacteria.

### GSDME

GSDME, also known as DFNA5 (deafness, autosomal dominant 5), was initially discovered to play a role in hearing impairment (Van Laer et al., 1998; Delmaghani et al., 2006). As a member of the gasdermin family, GSDME, like GSDMB and GSDMD, is highly expressed in the intestinal epithelium and functions as a pyroptosis-executing protein. However, GSDME is distinct from the other two proteins in that GSDME is not cleaved by caspase-1, -4, -6, -7, -8, or -9. Although caspase-3 has been used as a sign of apoptosis, a role in pyroptosis has also been identified. Stimulation by pro-apoptotic factors [such as tumor necrosis factor (TNF)] activates effector caspase-3 in the apoptotic pathway. Caspase-3 not only cleaves GSDMD to promote pyroptosis; but also activates GSDME, converting apoptosis into pyroptosis in cells with high expression levels of GSDME (Rogers et al., 2017).

GSDME-mediated pyroptosis is associated with GSDME expression levels. It has been shown that in cells with high GSDME levels, GSDME stimulation by chemotherapeutic drugs (apoptosis-inducing) can directly induce pyroptosis (Wang et al., 2017). In macrophages with low levels of GSDME expression, GSDME-dependent pyroptosis does not occur upon induction of apoptosis, even though GSDME is cleaved (Rogers et al., 2017; Lee et al., 2018; Broz et al., 2020). Like GSDMB (Zhou et al., 2020), GSDME is involved in Gzm-mediated cell death, a caspase-independent process. Killer-cell GZMB can also activate pyroptosis by direct cleavage of GSDME at the same site as caspase-3 (D270) (Zhang et al., 2020).

GSDME, a candidate tumor suppressor, affects cancers such as breast cancer, gastric cancer, melanoma, and colorectal cancer (Akino et al., 2007). Promoter DNA methylation inactivates GSDME in many cancer cells resulting in low levels of GSDME expression (Wang et al., 2013). The GSDME promotes tumor cell phagocytosis by macrophages and increases tumor-infiltrating natural killer and CD8^+^ T lymphocytes numbers and function (Wang et al., 2017).

GSDME-regulated pyroptosis promotes intestinal inflammation, which plays a role in the development of colitis-associated colorectal cancer (Tan et al., 2020). Significantly increased levels of GSDME protein were detected in the colonic mucosa of patients with IBD compared to healthy controls (Tan et al., 2020; Tan et al., 2021). In a mouse model of DSS-induced colitis, Gsdme-knockout mice exhibited reduced inflammation, and Gsdme−/− mice release higher levels of high mobility group box 1 (HMGB1) from IECs. Extracellular HMGB1 induces an inflammatory response (Boyapati et al., 2016), suggesting that GSDME-mediated pyroptosis promotes mucosal inflammation through the release of HMGB1 from IECs. GSDME deficiency effectively reduces HMGB1 release from colonic tissues, alleviating inflammation in a mouse model of DSS-induced colitis (Tan et al., 2020). Beyond HGMB1, recent studies have also suggested that GSDME-mediated pyroptosis releases pro-inflammatory cytokines (IL-1*β*, TNF-*α*, and IL-6) that contribute to CD pathogenesis (Tan et al., 2021).

While GSDME is important for antitumor immunity, not much research has been done on its role in chronic inflammation. GSDME has been shown to promote inflammation through the release of HGMB1 and other pro-inflammatory cytokines. GSDME-mediated pyroptosis is an important innate immunity process that protects the host against pathogenic infections, but whether it also plays a role in protecting the intestine from pathogenic bacteria remains unclear.

## DAMPs Released by Pyroptosis on IBD

Damaged cells released DAMPs that trigger an immune response. The release of DAMPs mainly includes passive release after rupture of the cytoplasmic membrane caused by cell death and active release from living cells represented by extracellular action (Murao et al., 2021). For positive release from living cells, because many types of DAMPs cannot be released *via* the typical protein secretion pathway consisting of the endoplasmic reticulum (ER) and the Golgi apparatus, DAMPs require the use of a carrier release, such as secretory lysosomes and exosomes, which are secreted by exocytosis (Gardella et al., 2002; Kim et al., 2021). Such danger signals can initiate an immune response by activating pattern recognition receptors (PRRs). Common PRRs include NOD-like receptors (NLRs), retinoic acid-inducible gene I (RIG-I)-like receptors (RLRs), C-type lectin receptors (CLRs), and multiple intracellular DNA sensors (Gong et al., 2020). In addition to PRRs, DAMPs can also be sensed by non-PRR DAMP receptors) and others (Ford and McVicar, 2009). DAMP-sensing receptors sense various DAMPs to exert inflammatory responses (Chen and Nuñez, 2010). Such inflammatory response benefits tissue repairing. However, excessive chronic inflammation is harmful to the host and leads to inflammatory diseases, including autoimmune diseases, IBD, and cancer (Gong et al., 2020; Roh and Sohn, 2018). Not surprisingly, a large number of DAMPs, including S100A calcium, High-mobility group box 1 (HMGB1), interleukin (IL)-1*α*, and IL-33, were found in patients with IBD (Boyapati et al., 2016). Fecal calprotectin testing has revolutionized IBD clinical practice with roles in differentiating IBD from functional gut disorders (Henderson et al., 2014). One consequence of this pore-forming activity is cytoplasmic swelling, which leads to the release of contents into the cytoplasm. The release of DAMPs by pyroptosis is key to its downstream consequences, allowing the propagation of inflammatory responses through multiple mechanisms. The non-selective pore-releasing DAMPs formed by pyroptosis contain HMGB1 (Broz et al., 2020), ASC specks (Baroja-Mazo et al., 2014), galectins (Russo et al., 2021), and mitochondria DNA (mtDNA) (de Vasconcelos et al., 2019), among other compounds.

### HGMB1

HMGB1 is a non-histone nuclear protein that translocates to the cytoplasm and is secreted into the extracellular space (Harris et al., 2012). The binding of HMGB1 to its receptors (including late glycosylation product receptors, TLR 4, and other receptors) stimulates cytokine secretion by macrophages and affects cell proliferation and migration (Fiuza et al., 2003). HMGB1 and inflammation occurrence are related (Andersson and Tracey, 2011). HGMB1 can be released through cellular scorch death, and this release is different from IL-1*β* release through the pore, where HGMB1 is dependent on cell lysis. When pyroptosis is blocked, HMGB1 is not released despite inflammasome activation and IL-1*β* secretion (Volchuk et al., 2020). Pyroptosis promotes acetylation of HGMB1 and facilitates the linkage of Cys23 and Cys45 by a disulfide bond, allowing released HGMB1 to elicit a robust inflammatory response (Nyström et al., 2013). Interestingly, HMGB1 is also thought to trigger inflammatory responses through macrophage endocytosis, causing lysosome rupture and Caspase-1 activation (Xu et al., 2014). Previous studies have indicated that HMGB1 is correlated with IBD, as well as with other inflammatory and autoimmune diseases (Harris et al., 2012). It can also be used as a biomarker to detect subclinical intestinal inflammation and mucosal healing in patients with IBD (Palone et al., 2014). GSDME-mediated pyroptosis promotes mucosal inflammation through the release of HMGB1 from IECs. Furthermore, deletion of GSDME effectively reduces HMGB1 expression and release in colonic tissue models and alleviates inflammation in mouse models of DSS-induced colitis (Tan et al., 2020).

### Galectin-1

Galectin-1 belongs to a family of proteins that bind *β*-galactosidase and which are involved in inflammation, allergy, and host-pathogen interaction (Camby et al., 2006). Previously studies have demonstrated that it plays pro-inflammatory roles in many diseases, including sepsis and osteoarthritis (Toegel et al., 2016; Lei et al., 2018). Galactin-1 is a common component of inflammatory cell death. Cytosolic LPS triggers pyroptosis regulated by GSDMD, releasing galectin-1 extracellularly. Galectin-1 plays several immunoregulatory roles and binds to CD45 on T cells to attenuate phosphatase activity (Earl et al., 2010; Sundblad et al., 2017). Detection of intracellular LPS drives the release of galectin-1 from immune and non-immune cells, after which galectin-1 binds to the receptor CD45, likely limiting phosphatase activity and reducing the inhibitory effect of this receptor on inflammatory responses (Russo et al., 2021).

In addition to HGMB1 and galectin-1 (Russo et al., 2021), DAMPs such as mtDNA are also released by pyroptosis. However, it is unknown whether these molecules contribute to intestinal inflammation.

### IL-1*β*

Cellular receptors recognize PAMPs and DAMPs, which activate pro-inflammatory caspases like caspase-1. Activated caspase-1 cleaves GSDMD, causing cell membrane pores to form. As the inflammatory response proceeds, more and more GSDMD is cleaved, and additional pores are formed, ultimately leading to membrane rupture. GSDMD pores have an inner diameter of 10–20 nm (Shi et al., 2017; Mulvihill et al., 2018; Ruan et al., 2018), allowing the release of mature pro-inflammatory cytokine into the extracellular space (Liu et al., 2016). pro- IL-1*β* and IL-18 are cleaved by activated caspase-1 to form mature IL-1*β* and IL-18, which are then released into the extracellular region *via* pores (Evavold et al., 2018; Heilig et al., 2018).

Unlike caspase-1, which is activated by classical inflammasomes, caspase-4/5/11 are activated by non-classical inflammasome pathways and bind directly to LPS from Gram-negative bacteria (Shi et al., 2014). While caspase-4/5/11 does not directly process pro-IL-1*β* and pro-IL-18, non-selective membrane channels formed by caspase-4/5/11-induced pyroptosis lead to potassium efflux, which induces NLRP3 inflammasome assembly (canonical inflammasome pathway) and IL-1*β*/IL-18 maturation (Kayagaki et al., 2011; Rühl and Broz, 2015; Cunha et al., 2017). GSDMD pores are large enough to allow passage of mature IL-1*β* and appear to be necessary for the release of activated IL-1*β* (Mitra and Sarkar, 2019). IL-1*β* does not carry a signal peptide sequence and is activated by caspase-1 (Cerretti et al., 1992), then released outside of the cell through pores formed by pyroptosis. IL-1*β* is induced by NLRP3 inflammasomes in myeloid cells, where it acts to enhance intestinal inflammation by accumulating innate lymphoid cells (ILCs) and T-helper 17 (TH17) cells (Seo et al., 2015). The IL-1*β* release is thought to be cell lysis dependent and a by-product of the non-selective release of cell contents (Shi et al., 2017). However, an increasing number of studies have reported that IL-1*β* release in a variety of cell types (e.g., neutrophils) is not dependent on cell lysis (Dinarello, 2010; Bakele et al., 2014; Monteleone et al., 2018). Mature IL-1*β* is secreted in the absence of lysis of cells. Several reports have confirmed that IL-1*β* can be secreted by vesicles (Chen et al., 2014).

IL-1*β* is a typical pro-inflammatory cytokine involved in the inflammatory process, activating immune cells to perform anti-microbial and pro-inflammatory functions (Dinarello, 2010). Increased levels of IL-1*β* can be detected in intestinal lesions and mucosal cells of patients with IBD (Ligumsky et al., 1990). This seems to suggest that IL-1*β* has a pro-inflammatory effect. However, no clinical trials have shown definitive efficacy of IL-1*β* inhibition in patients with IBD. Its role in colitis remains controversial. The following are a few examples of this confusing phenomenon. Mice deficient in IL-1*β* exhibited more aggressive intestinal inflammation compared to controls, suggesting a potential protective effect of IL-1*β* (Bersudsky et al., 2014); Exogenous IL-1*β* is protective against acute colitis in Gsdmd−/− mice (Gao et al., 2021). These indicate another function of IL-1*β* --- as a protector. Increased GSDMD transcripts were observed in locally inflamed intestinal mucosal tissue of IBD patients, and GSDMD is involved in non-soluble IL-1*β* release, whether this is responsible for the persistence of intestinal inflammation also needs to be further investigated. More in-depth studies are needed on the role of IL-1*β* in IBD, and the factors that determine its function may involve the mode of stimulation and release.

### IL-18

Like IL-1*β*, IL-18 plays a key role in intestinal homeostasis and inflammation as a member of the IL-1 family (Lopetuso et al., 2013; Neurath, 2014). IL-18 binds to the IL-18 receptor and, upon binding, triggers receptor heterodimerization and assembly of an intracellular Myd88 signaling platform (Adachi et al., 1998). In addition, IL-18 induces the production of IFN-*γ*, an important mediator of antiviral and antibacterial immunity (Man et al., 2017). As a pro-inflammatory cytokine, IL-18 has emerged as an important player in host-microbe interactions, and it has been proposed to be a key factor in IBD (Elinav et al., 2011). Caspase-4/11 mediates the secretion of IL-18 intestinal epithelial defenses against *Salmonella Typhimurium* (*S. Typhimurium* infection) (Knodler et al., 2014). IECs resistant to colitis produces antimicrobial peptides (AMPs) when they are stimulated by IL-18 processed by the NLRP6 inflammasome (Levy et al., 2015). Nevertheless, a recent study showed that IL-18 is directly responsible for promoting goblet cell dysfunction during colitis, leading to mucosal barrier breakdown by inhibiting goblet cell maturation (Nowarski et al., 2015). As with IL-1*β*, its role in colitis remains controversial. Administration of exogenous recombinant IL-18 attenuates colitis in mice with inflammasome defects, further supporting the protective role of IL-18 in colitis (Oficjalska et al., 2015). However, on the other hand, IL-18 is thought to promote the ability of colitis by inducing inflammatory mediators such as TNF*α* and chemokines (Sivakumar et al., 2002), inhibition of IL-18 has also been shown to trigger protection in experimental colitis, supporting the role of IL-18 in exacerbating colitis (Siegmund et al., 2001). Increased epithelial and hematopoietic IL-18 expression and cytokines in patients with IBD (Monteleone et al., 1999). Deletion of IL-18 receptor from intestinal epithelial cells IL-18R protects mice from DSS-induced colitis (Nowarski et al., 2015). Taken together, IL-1*β* and IL-18 defend against bacterial infection and help maintain intestinal homeostasis, but aggravate chronic intestinal inflammation.

## Conclusion and Future Perspectives

**FIGURE 3:**
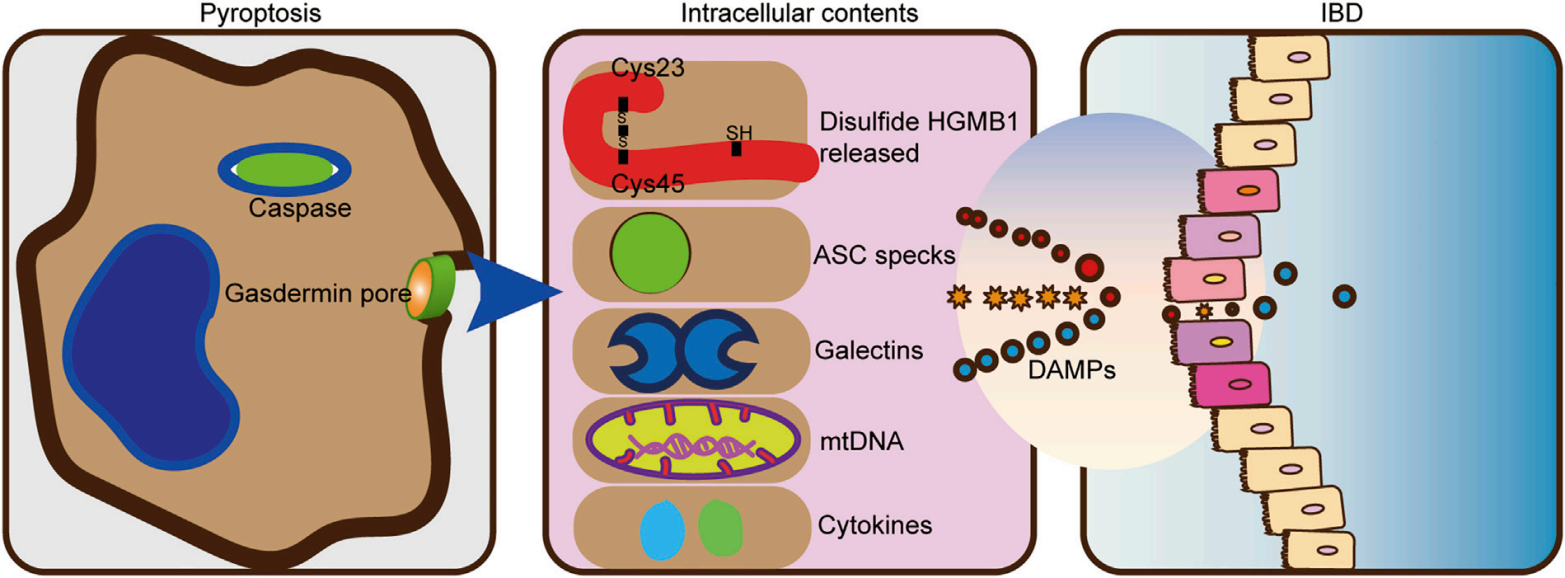
DAMPs from pyroptosis drive intestinal inflammation. The pro-inflammatory cytokines IL-1*β* and IL-18 are cleaved and secreted. HMGB1, mtDNA, and numerous damage-associated molecular patterns are also released by pyroptosis. The release of endogenous damage-associated molecular patterns (DAMPs) can further trigger an inflammatory response in IBD.

IBD is a common intestinal disease with complex pathogenesis that involves genetic, environmental, epithelial, microbial, and immune factors. Both inflammasomes and caspases are involved in pyroptosis. Moreover, their effects on IBD have been more extensively studied, piquing our curiosity about the role of pyroptosis in IBD. Pyroptosis is a form of pro-inflammatory cell death in which gasdermin proteins are cleaved, after which cytotoxic domains bind to cell membrane to form pores that release cytokines and other DAMPs. IL-1*β* and IL-18 function to defend against bacterial infection and maintain intestinal homeostasis, but also aggravate chronic intestinal inflammation. It is generally assumed that DAMPs released by pyrogenesis drive intestinal inflammation (Figure 3). However, the DAMPs released by pyroptosis are very complex. It is not clear whether other DAMPs also contribute to intestinal inflammation, and the exact roles of these DAMPs require further clarification.

To investigate the relationship between pyroptosis and IBD, we focused on GSDMB, GSDMD, and GSDME (which have been identified as being involved in the mechanism of pyroptosis), members of the well-studied gasdermin protein family. GSDMB is a gene associated with susceptibility to IBD, but the mechanism by which it regulates intestinal inflammation is not well understood. In contrast, GSDMD is the most studied gasdermin protein, but it has been reported to exert opposite effects in IECs and macrophages. Finally, GSDME has been found to promote intestinal inflammation through the release of HGMB1 and cytokines. Given that gasdermin proteins and DAMPs are closely associated with pyroptosis and play important roles in inflammation, many unanswered questions remain, and these unexplored areas may be promising areas for future research.
